# Supplementary material for: Drug repurposing for aging research using model organisms
Source: Aging Cell. 2017 Jun 16;16(5):1006–15. doi: 10.1111/acel.12626 (PMC5595691; doi:10.1111/acel.12626)
Supplement: Supplementary file 7 — Data S1 Zip‐Archive of all report cards. [file ACEL-16-1006-s007.zip › RC_ALE.pdf]

ALE

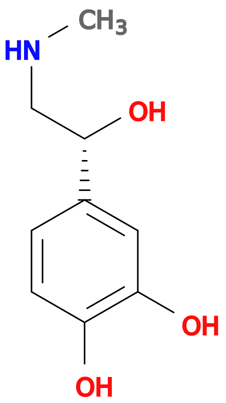

Database identifiers

ChEMBLCompound	CHEMBL679
ChEMBLCompound	CHEMBL42280
DrugBank	DB00668
CHEBI	28918
eMolecules	475028

Ranking

	Rank	Score
Drosophila	398/697	0.365
C. elegans	247/591	0.223

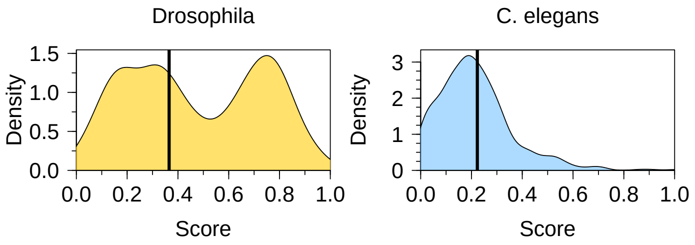

	Ageing implication	Domain conservation	Binding site conservation	Binding affinity	Bioavailability	Lipinski	Promiscuity	Purchasability	Drug approval	**Total**
Drosophila	1.0	0.922	0.567	0.353	(0.9)	0.0	-0.0	0.1	0.1	0.365
C. elegans	1.0	0.748	0.422	0.353	0.212	0.0	-0.0	0.1	0.1	0.223

Names

- **(R)-adrenaline**
- (-)-3,4-dihydroxy-alpha-((methylamino)methyl)benzyl alcohol
- (R)-(-)-Adnephrine
- (R)-(-)-Epirenamine
- 4-(1R)-1-Hydroxy-2-(methylamino)ethyl-1,2-benzenediol
- Adrenaclick
- Ana-Kit
- Auvi-Q
- Bronkaid Mist
- Epi E Z Pen Jr
- Epinephrin
- Epipen

· Levo-Methylaminoethanolcatechol
· levoepinephrine
· Primatene Mist
· Sus-Phrine
· Twinject

Roles

adrenergic agonist, vasodilator agent, mydriatic agent, bronchodilator agent, alpha-adrenergic agonist, vasoconstrictor agent, beta-adrenergic agonist, sympathomimetic agent, hormone, Adrenergic receptor agonist

Status

Approved drug (according to ChEMBL)	Yes
Administration Route	Intramuscular, Oral, Intravenous, Nasal, Respiratory (inhalation), Ophthalmic
Number of Rule of 5 violations	0
Binding affinity to original target in log units (RF-Score prediction)	4.39
Burns *C. elegans* bioavailability prediction	-5.45

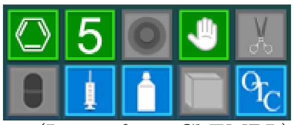
(Image from ChEMBL)

Medical Information

Indicatation: Used to treat anaphylaxis and sepsis. Also one of the body's main adrenergic neurotransmitters.
Mechanism of action: Epinephrine works via the stimulation of alpha and beta-1 adrenergic receptors, and a moderate activity at beta-2 adrenergic receptors.
Toxicity: Skin, LD50 = 62 mg/kg (rat)
Metabolism: Epinephrine is rapidly inactivated in the body and is degraded by enzymes in the liver and other tissues. The larger portion of injected doses is excreted in the urine as inactivated compounds and the remainder either partly unchanged or conjugated. The drug becomes fixed in the tissues and is inactivated chiefly by enzymatic transformation to metanephrine or normetanephrine either of which is subsequently conjugated and excreted in the urine in the form of sulfates and glucuronides. Either sequence results in the formation of 3-methoxy-4-hydroxy-mandelic acid which also is detectable in the urine. Main metabolic enzymes include MAO and COMT
Retail: Adamis Laboratories, Amend, American Regent, Amphastar Pharmaceuticals, APP Pharmaceuticals, Armstrong Pharmaceuticals Inc. and 49 more suppliers
Primatene mist inhaler - 1.0 USD / ml
Epinephrine 0.1 mg/ml syringe - 1.32 USD / ml
Adrenalin cl 1 mg/ml vial - 1.42 USD / ml
Epinephrine 1 mg/ml ampul - 2.9 USD / ml
Adrenalin 1 mg/ml ampul - 2.93 USD / ml
Epinephrine bitartrate crys - 4.2 USD / g
and 27 more options

(Information from DrugBank)

Compound Target Characteristics

Superoxide dismutase [Cu-Zn]

Best gene implication in ageing for this target family came from gene P61851 annotated in UniProt release 2014_02. Annotation GO 8340 (determination of adult lifespan) was Inferred from Mutant Phenotype

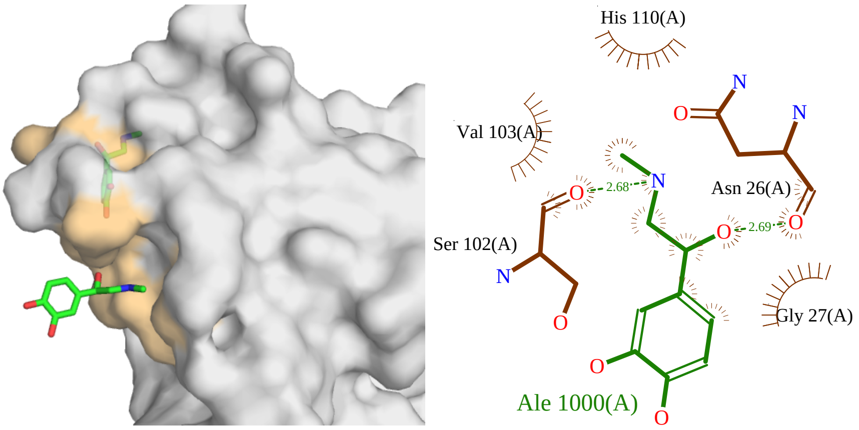

protein	amino acids contacts (binding site)												
PDB:4a7u:chainA:P00441	E	Q	K	N	G	P	V	K	W	E	S	V	H
sp:P00441:SODC_HUMAN	E	Q	K	N	G	P	V	K	W	E	S	V	H
sp:P07632:SODC_RAT	E	Q	K	G	E	P	V	V	S	E	R	V	H
tr:Q6LDS4:Q6LDS4_RAT	E	Q	K	G	E	P	V	V	S	E	R	V	H
sp:P08228:SODC_MOUSE	E	Q	K	G	E	P	V	V	S	E	R	V	H
sp:P61851:SODC_DROME	E	Q	E	G	T	P	V	K	S	T	S	K	D
tr:G5ECR5:G5ECR5_CAEEL	D	Q	-	G	S	F	L	K	N	S	S	L	Y
sp:P34461:SODE_CAEEL	D	Q	-	G	S	F	L	K	N	S	S	L	Y
sp:P00445:SODC_YEAST	E	Q	A	S	E	P	T	T	S	K	S	L	T

	whole protein		domain-based		contact-based	
protein	ident	simil	ident	simil	ident	simil
PDB:4a7u:chainA:P00441	0.99	1.0	0.99	1.0	1.0	1.0
sp:P00441:SODC_HUMAN	1.0	1.0	1.0	1.0	1.0	1.0
sp:P07632:SODC_RAT	0.83	0.94	0.83	0.94	0.62	0.56
tr:Q6LDS4:Q6LDS4_RAT	0.82	0.93	0.82	0.93	0.62	0.56
sp:P08228:SODC_MOUSE	0.84	0.94	0.83	0.94	0.62	0.56
sp:P61851:SODC_DROME	0.61	0.84	0.6	0.85	0.46	0.57
tr:G5ECR5:G5ECR5_CAEEL	0.35	0.57	0.44	0.71	0.23	0.42
sp:P34461:SODE_CAEEL	0.35	0.57	0.44	0.71	0.23	0.42
sp:P00445:SODC_YEAST	0.54	0.83	0.55	0.84	0.31	0.55

Sod (FBgn0003462) associated phenotypes

calorie restriction conditional, chemical conditional, chemical sensitive, heat sensitive, increased cell number, locomotor behavior defective, long lived, male limited, oxidative stress response defective, partially, partially lethal - majority die, progressive, radiation sensitive, reduced, short lived

(Information from FlyBase)

Sod (UniProt:P61851) annotation

Function: Destroys radicals which are normally produced within the cells and which are toxic to biological systems.
Cofactor:Cu cationNote=Binds 1 copper ion per subunit.;
Cofactor: Zn(2+)Note=Binds 1 zinc ion per subunit.;
Subunit: Homodimer.
Subcellular location: Cytoplasm.

(Information from UniProt)

sod-4 (UniProt:G5ECR5) annotation

Function: Destroys radicals which are normally produced within the cells and which are toxic to biological systems. (RuleBase:RU000393).

Cofactor: Cu cation Evidence=(RuleBase:RU000393); Note=Binds 1 copper ion per subunit. (RuleBase:RU000393);

Cofactor: Zn(2+) Evidence=(RuleBase:RU000393); Note=Binds 1 zinc ion per subunit. (RuleBase:RU000393);

(Information from UniProt)

sod-4 (UniProt:P34461) annotation

Function: Destroys radicals which are normally produced within the cells and which are toxic to biological systems.

Cofactor:Cu cationNote=Binds 1 copper ion per subunit. ;

Cofactor: Zn(2+)Note=Binds 1 zinc ion per subunit. ;

Subcellular location: Isoform 1: Secreted, extracellular space.

Subcellular location: Isoform 2: Membrane ; Peripheral membrane protein

Tissue specificity: Isoform 2 is preferentially expressed in eggs.

(Information from UniProt)
